# Hepatic arterial infusion chemotherapy combined with anti-PD-1/PD-L1 immunotherapy and molecularly targeted agents for advanced hepatocellular carcinoma: a real world study

**DOI:** 10.3389/fimmu.2023.1127349

**Published:** 2023-04-26

**Authors:** Weihao Zhang, Kai Zhang, Changfu Liu, Wei Gao, Tongguo Si, Qiang Zou, Zhi Guo, Xueling Yang, Mei Li, Dongming Liu, Han Mu, Huikai Li, Haipeng Yu, Wenge Xing

**Affiliations:** ^1^ Department of Interventional Therapy, Tianjin Medical University Cancer Institute & Hospital, National Clinical Research Center for Cancer, Tianjin, China; ^2^ Tianjin’s Clinical Research Center for Cancer, Tianjin Medical University Cancer Institute & Hospital, Tianjin, China; ^3^ Key Laboratory of Cancer Prevention and Therapy, Tianjin Medical University Cancer Institute & Hospital, Tianjin, China; ^4^ Department of Hepatobiliary, Tianjin Medical University Cancer Institute & Hospital, National Clinical Research Center for Cancer, Tianjin, China

**Keywords:** hepatic arterial infusion chemotherapy, molecularly targeted agents, advanced hepatocellular carcinoma, conversion surgery, survival benefit, anti-PD-1/PD-L1 immunotherapy

## Abstract

**Background:**

Molecular targeted therapy combined with immunotherapy significantly improves the prognosis of patients with advanced liver cancer. Additionally, hepatic arterial infusion chemotherapy (HAIC) can improve the prognosis of patients with advanced liver cancer. This real-world study aimed to evaluate the clinical efficacy and safety of HAIC combined with molecular targeted therapy and immunotherapy in the treatment of primary unresectable hepatocellular carcinoma (uHCC).

**Methods:**

A total of 135 patients with uHCC were enrolled in this study. Progression-free survival (PFS) was the primary endpoint. The efficacy of the combination therapy was assessed based on the modified Response Evaluation Criteria in Solid Tumors (mRECIST) guidelines. Overall survival (OS), adverse events (AEs) and surgical conversion rate were the secondary endpoints. Univariate and multivariate Cox regression analyses were performed to examine independent prognostic factors. For sensitivity analysis, inverse probability weighting (IPW) was used to balance the influence of the tested confounding factors between groups to verify the robustness of conversion surgery for survival benefits. The E-values were estimated to assess robustness to unmeasured confounders.

**Results:**

The median number of therapies was three. Approximately 60% of the patients had portal vein tumour thrombosis (PVTT). The most common targeted drugs were lenvatinib and bevacizumab, whereas the most common immunotherapy drug was sintilimab. The overall objective response rate (ORR) was 54.1%, and the disease control rate (DCR) was 94.6%. A total of 97 (72%) patients experienced AEs of grades 3–4. Fatigue, pain and fever were the most common symptoms of grade 3-4 AEs. The median PFS was 28 months and 7 months in the successful and unsuccessful conversion groups, respectively. The median OS was 30 months and 15 months in the successful and unsuccessful conversion groups, respectively. Successful conversion surgery, sex, hapatic vein invasion, BCLC stage, baseline tumour size, AFP levels and maximum therapeutic response were independent prognostic factors for PFS. Successful conversion surgery, number of interventions, hapatic vein invasion and total bilirubin levels were independent prognostic factors for OS. After IPTW, no standardised differences exceeding 0.1 were found. IPW-adjusted Kaplan–Meier curves showed that successful conversion surgery was an independent prognostic factor for both PFS and OS. The E-values of successful conversion surgery were 7.57 and 6.53 for OS and PFS, respectively, which indicated a relatively robust impact of successful conversion surgery on the prognosis of patients.

**Conclusion:**

Patients with primary uHCC undergoing HAIC combined with immunotherapy and molecular targeted therapy have a higher tumour regression rate and the side effects are manageable. Patients undergoing surgery after combination therapy have survival benefits.

## Introduction

On a global scale, liver cancer ranks fifth among malignant cancers (1). The most common subtype of primary liver cancer is hepatocellular carcinoma (HCC) (2). The main treatment strategies for early-stage HCC include surgical resection, ablation and liver transplantation (3). However, most patients with HCC are diagnosed at an advanced stage and hence have a poor prognosis. In recent years, the treatment of advanced HCC has rapidly evolved with the introduction of novel systemic therapies. The IMbrave 150 study showed that compared with sorafenib, atezolizumab combined with bevacizumab had better therapeutic effects in patients with unresectable hepatocellular carcinoma (uHCC) (4). After sorafenib, the combination of atezolizumab and bevacizumab was the first therapeutic strategy that demonstrated promising results in a randomised controlled trial. At present, the first-line treatment for advanced liver cancer is a combination of molecular targeted drugs and immune checkpoint inhibitors (5).

As systemic therapy advances, indications for TACE therapy in HCC are narrowing. The Barcelona Clinic Liver Cancer (BCLC) staging system is the most commonly used system for classifying HCC stages (6). In the previous BCLC guidelines, TACE was recommended as the primary treatment method for BCLC B-stage HCC. However, the 2022 BCLC staging system has re-categorised the stratification of risk for patients with B-stage HCC. The recommended first-line treatment for patients with BCLC B-stage HCC with diffuse, infiltrative and extensive bilobar liver involvement has been changed from TACE to systemic therapy (5). We speculate that the reason for this change, in addition to the advancement of systemic therapy, is related to the poor efficacy of TACE therapy in patients with moderate and high tumour burdens (7, 8).

Hepatic arterial infusion chemotherapy (HAIC) is a locoregional therapy that involves the administration of chemotherapeutic agents at higher concentrations directly into tumour sites *via* tumour-associated arterial branches (9). A recent randomised controlled trial showed that compared with TACE, FOLFOX-HAIC significantly improved overall survival (OS) and progression-free survival (PFS) in patients with unresectable large tumours (10). The effectiveness of HAIC has also been confirmed in a real-world study (11). Considering that both HAIC and systemic therapy are effective for patients with moderate and high tumour burdens, the therapeutic efficacy of the combination of these two therapies is currently undergoing investigation. Compared with sorafenib monotherapy, combination therapy with sorafenib and FOLFOX-HAIC can improve the objective response rate (ORR) and OS of patients with HCC (12, 13). In addition, the combination of HAIC, anti-PD-1-based immunotherapy and molecularly targeted agents may improve patient outcomes (14, 15). However, relevant previous studies reporting on the abovementioned combination therapeutic strategies had a limited number of participants, which limits the external validation of the results. To the best of our knowledge, no large-scale, real-world studies have reported the therapeutic effects of HAIC combined with systemic therapy. Therefore, this study aimed to evaluate the safety and effectiveness of HAIC combined with immunotherapy and molecular targeted therapy in patients with advanced HCC.

## Materials and methods

### Patients

This retrospective study was conducted in accordance with the principles outlined by the Declaration of Helsinki (16) and was approved by the Tianjin Medical University Cancer Institute and Hospital Review Board (No.: bc2020099). A random number was assigned to each participant, and other information that could reveal the identity of participants was removed.

**Figure 1:**
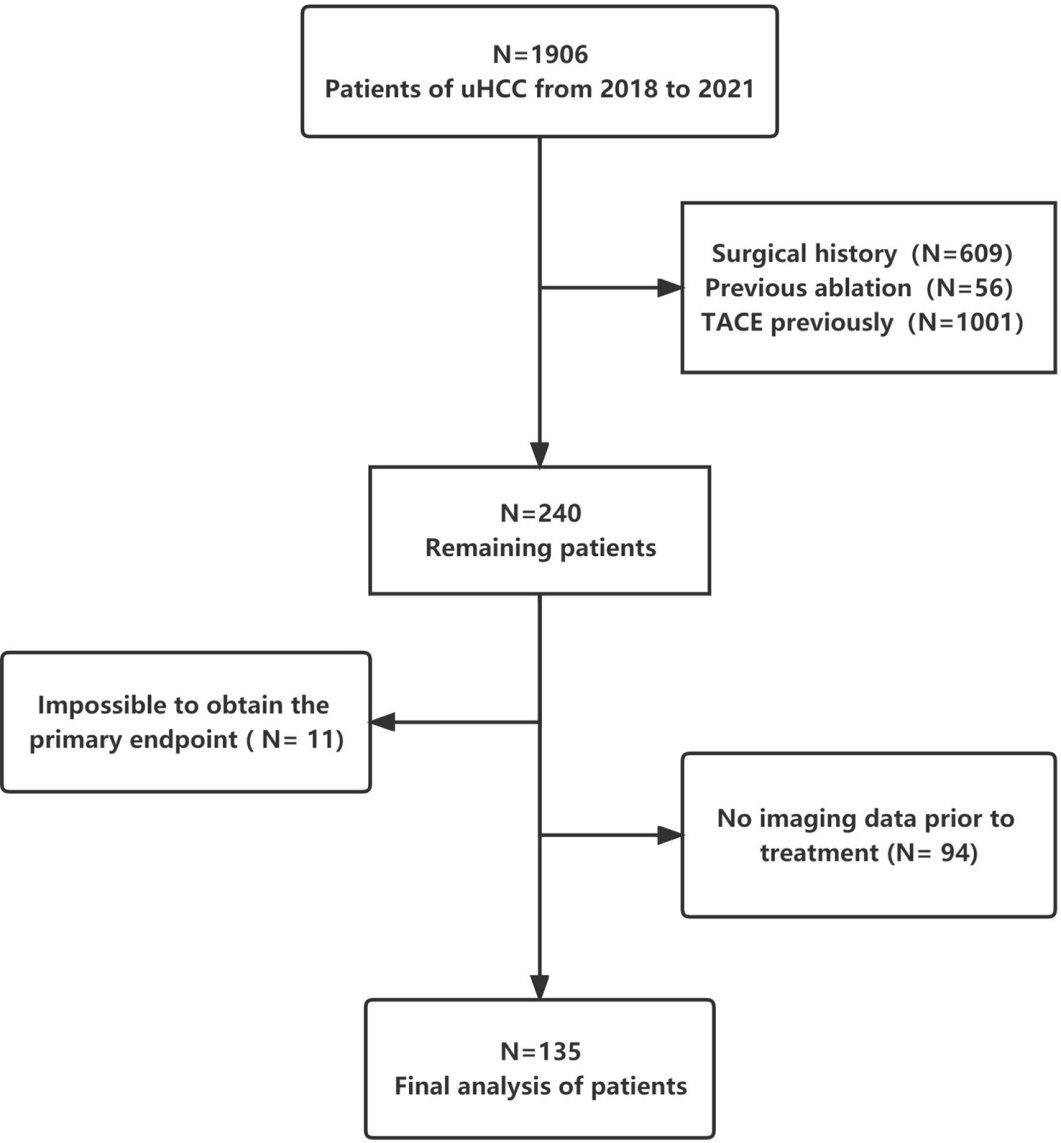
Flowchart demonstrating the patient inclusion strategy.

Patients with uHCC who received triple therapy (HAIC + anti-PD-1-based immunotherapy + molecular targeted therapy) between November 2018 and December 2021 at Tianjin Medical University Cancer Institute and Hospital were enrolled. All patients were evaluated by our multidisciplinary board for surgical resection. The board comprises multidisciplinary specialists, including hepatobiliary surgeons, oncologists, interventionists, radiologists and radiotherapy physicians. To increase the sample size and ensure representativeness, there was no limitation regarding the specific use of immune checkpoint inhibitors and targeted drugs in the included patients. **Figure 1** demonstrates a flowchart representing the patient enrolment strategy with detailed inclusion and exclusion criteria.

### Treatment protocol

Patients were locally anaesthetised, and the Seldinger technique was used to puncture the femoral artery. To examine blood supply at the tumour site, digital subtraction angiography was used to visualise the anatomical features of the celiac, superior mesenteric and hepatic arteries. HAIC was conducted using a 2.7-F microcatheter placed in the tumour-feeding arteries. The FOLFOX regimen was used as follows: 4-hour infusion of 85-mg/m^2^ oxaliplatin, 2–3-hour infusion of 400-mg/m^2^ calcium folinate and a bolus injection of 400-mg/m^2^ fluorouracil, followed by 23-hour infusion of 1200-mg/m^2^ fluorouracil on day 1 of treatment. Drug doses may be adjusted depending on the Child–Pugh grade and chemotherapy tolerance. HAIC was repeated every 4–6 weeks until disease progression or unacceptable toxicity was observed or the treatment plan was changed. Treatment may be interrupted or dose adjustments may be required if toxicity is intolerable. When grade 3 or 4 adverse events occur, oxaliplatin would be reduced to 65 mg/m^2^, and 5-fluorouracil to 300 mg per bolus and 1000 mg per cycle respectively.

### Anti-PD-1-based immunotherapy and molecular targeted therapy

Before or after the first HAIC session, patients were intravenously administered anti-PD-1 antibodies every 3 weeks (200-mg sintilimab, 200-mg tislelizumab, 200-mg camrelizumab, 240-mg toripalimab or 200-mg pembrolizumab). For anti-angiogenesis treatment, the patients were administered 8-mg lenvatinib orally once daily, 200-mg sorafenib orally twice daily and 250-mg apatinib orally once daily. Patients in the T + A group were intravenously administered atezolizumab (1200 mg) plus bevacizumab (15 mg/kg) or sintilimab (200 mg) plus bevacizumab biosimilar (IBI305) every 3 weeks.

### Primary endpoints and data collection

PFS was the primary endpoint. Therapeutic efficacy was evaluated based on the modified Response Evaluation Criteria in Solid Tumors (mRECIST) (17). PFS was defined as the time from initiation of treatment to the end of progression or death. The secondary endpoints included OS, ORR (complete response [CR] + partial response [PR]), disease control rate (DCR; ORR + stable disease [SD]), surgical conversion rate and adverse events (AEs) defined by the Common Terminology Criteria for Adverse Events, version 4.0.

### Statistical analysis

Categorical variables were analysed *via* the Fisher’s exact test and were expressed as numbers (percentages). Data with normal distribution were analysed using the t-test or ANOVA analysis. Data with non-normal distribution were analysed using the rank sum test and were expressed as the median (interquartile range [IQR]). Survival was estimated using the Kaplan–Meier method, and the data were analysed *via* univariate and multivariate Cox proportional risk regression analyses. A p-value of <0.05 was considered significant.

Sensitivity analysis was conducted using two approaches. First, inverse probability weighting (IPW) was employed to manage potential imbalance among covariates between two groups (18). Second, E-values were estimated to examine unmeasured confounders owing to the observational study design (19) and evaluate the impact of unmeasured confounders on the outcomes of patients. Statistical analysis was performed using the R (version 4.2.1) software.

## Result

### Characteristics of patients and tumors

A total of 2019 patients were screened between November 2018 and December 2021; of which, 135 met the eligibility criteria for inclusion. A flowchart demonstrating the patient selection strategy and inclusion and exclusion criteria is shown in **Figure 1**. A majority of patients (82.2%) were men. The most prevalent cause of underlying liver disease was chronic hepatitis B virus infection (93.3%). More than half of the patients (58.8%) had portal vein tumour thrombosis. The median tumour size was 8.95 cm (IQR = 6.27–13.0 cm). Lenvatinib and bevacizumab were the most common targeted drugs, whereas sintilimab was the most common immunotherapeutic drug. Patients were divided into two groups based on whether conversion surgery was successful (**Table 1**). The median number of HAIC sessions was 3 (IQR = 2–3). The median number of HAIC sessions was higher in the successful conversion surgery group than in the unsuccessful conversion surgery group. However, the difference was not significant.

**Table 1:** Baseline information of patients.

	[ALL] *N=135*	No *N=95*	Yes *N=40*	p.overall
Sex				0.848
Female	24 (17.8%)	16 (16.8%)	8 (20.0%)	
Male	111 (82.2%)	79 (83.2%)	32 (80.0%)	
Age	58.0 [51.0;64.5]	57.0 [50.0;64.0]	59.0 [54.0;66.2]	0.240
Number of interventions	3.00 [2.00;3.00]	2.00 [1.00;3.50]	3.00 [2.00;3.00]	0.311
Targeted drug				0.112
Apatinib	5 (3.70%)	2 (2.11%)	3 (7.50%)	
Bevacizumab	57 (42.2%)	36 (37.9%)	21 (52.5%)	
Lenvatinib	67 (49.6%)	52 (54.7%)	15 (37.5%)	
Sorafenib	6 (4.44%)	5 (5.26%)	1 (2.50%)	
ICI				0.460
Atezolizumab	1 (0.74%)	1 (1.05%)	0 (0.00%)	
Camrelizumab	31 (23.0%)	24 (25.3%)	7 (17.5%)	
Pembrolizumab	1 (0.74%)	1 (1.05%)	0 (0.00%)	
Sintilimab	96 (71.1%)	65 (68.4%)	31 (77.5%)	
Tislelizumab	3 (2.22%)	3 (3.16%)	0 (0.00%)	
Toripalimab	3 (2.22%)	1 (1.05%)	2 (5.00%)	
Hypertension				0.853
No	101 (74.8%)	72 (75.8%)	29 (72.5%)	
Yes	34 (25.2%)	23 (24.2%)	11 (27.5%)	
Diabetes				0.777
No	119 (88.1%)	83 (87.4%)	36 (90.0%)	
Yes	16 (11.9%)	12 (12.6%)	4 (10.0%)	
Heart disease				0.669
No	129 (95.6%)	90 (94.7%)	39 (97.5%)	
Yes	6 (4.44%)	5 (5.26%)	1 (2.50%)	
Smoking				0.028
No	99 (73.3%)	64 (67.4%)	35 (87.5%)	
Yes	36 (26.7%)	31 (32.6%)	5 (12.5%)	
Liver etiology				0.278
Alcohol	1 (0.74%)	0 (0.00%)	1 (2.50%)	
HBV	126 (93.3%)	90 (94.7%)	36 (90.0%)	
HCV	8 (5.93%)	5 (5.26%)	3 (7.50%)	
Hepatic vein invasion				0.634
No	113 (86.3%)	78 (84.8%)	35 (89.7%)	
Yes	18 (13.7%)	14 (15.2%)	4 (10.3%)	
Portal vein tumor thrombus (vp)				0.489
0	54 (41.2%)	37 (40.2%)	17 (43.6%)	
2	6 (4.58%)	3 (3.26%)	3 (7.69%)	
3	29 (22.1%)	23 (25.0%)	6 (15.4%)	
4	42 (32.1%)	29 (31.5%)	13 (33.3%)	
BCLC				0.204
A	9 (6.92%)	4 (4.40%)	5 (12.8%)	
B	22 (16.9%)	14 (15.4%)	8 (20.5%)	
C	98 (75.4%)	72 (79.1%)	26 (66.7%)	
D	1 (0.77%)	1 (1.10%)	0 (0.00%)	
Baseline tumor size	8.95 [6.27;13.0]	10.0 [7.03;14.0]	7.95 [5.38;10.7]	0.045
Maximum Efficacy Evaluation				0.089
CR	1 (0.90%)	0 (0.00%)	1 (1.37%)	
PR	59 (53.2%)	34 (46.6%)	25 (65.8%)	
SD	45 (40.5%)	32 (43.8%)	13 (34.2%)	
PD	6 (5.41%)	6 (8.22%)	0 (0.00%)	
WBC	5.36 [4.17;6.88]	5.29 [3.96;7.18]	5.59 [4.51;6.39]	0.810
PLT	165 [118;243]	163 [118;230]	170 [117;247]	0.816
PT	12.3 [11.7;13.1]	12.5 [11.7;13.2]	12.1 [11.7;12.8]	0.203
APTT	27.7 [25.6;30.0]	27.9 [25.9;30.3]	27.5 [25.1;29.5]	0.326
GLU	5.12 [4.53;5.93]	5.18 [4.53;6.07]	5.07 [4.58;5.49]	0.874
SCR	64.0 [56.5;76.0]	64.0 [56.5;75.5]	67.0 [56.5;76.0]	0.320
ALB	39.1 [35.6;42.0]	38.4 [35.1;41.8]	40.5 [37.5;42.6]	0.024
ALT	32.0 [20.0;52.0]	33.0 [20.0;52.0]	32.0 [19.2;49.2]	0.544
AST	52.0 [34.0;85.0]	59.0 [35.0;92.0]	45.0 [33.8;65.2]	0.104
TBIL	17.1 [12.1;23.7]	18.2 [12.2;25.2]	15.1 [11.4;21.3]	0.117
AFP team (400ng/ml)				0.092
High	68 (52.3%)	52 (57.8%)	16 (40.0%)	
Low	62 (47.7%)	38 (42.2%)	24 (60.0%)	

### Short-term efficacy and side effects

**Figure 2:**
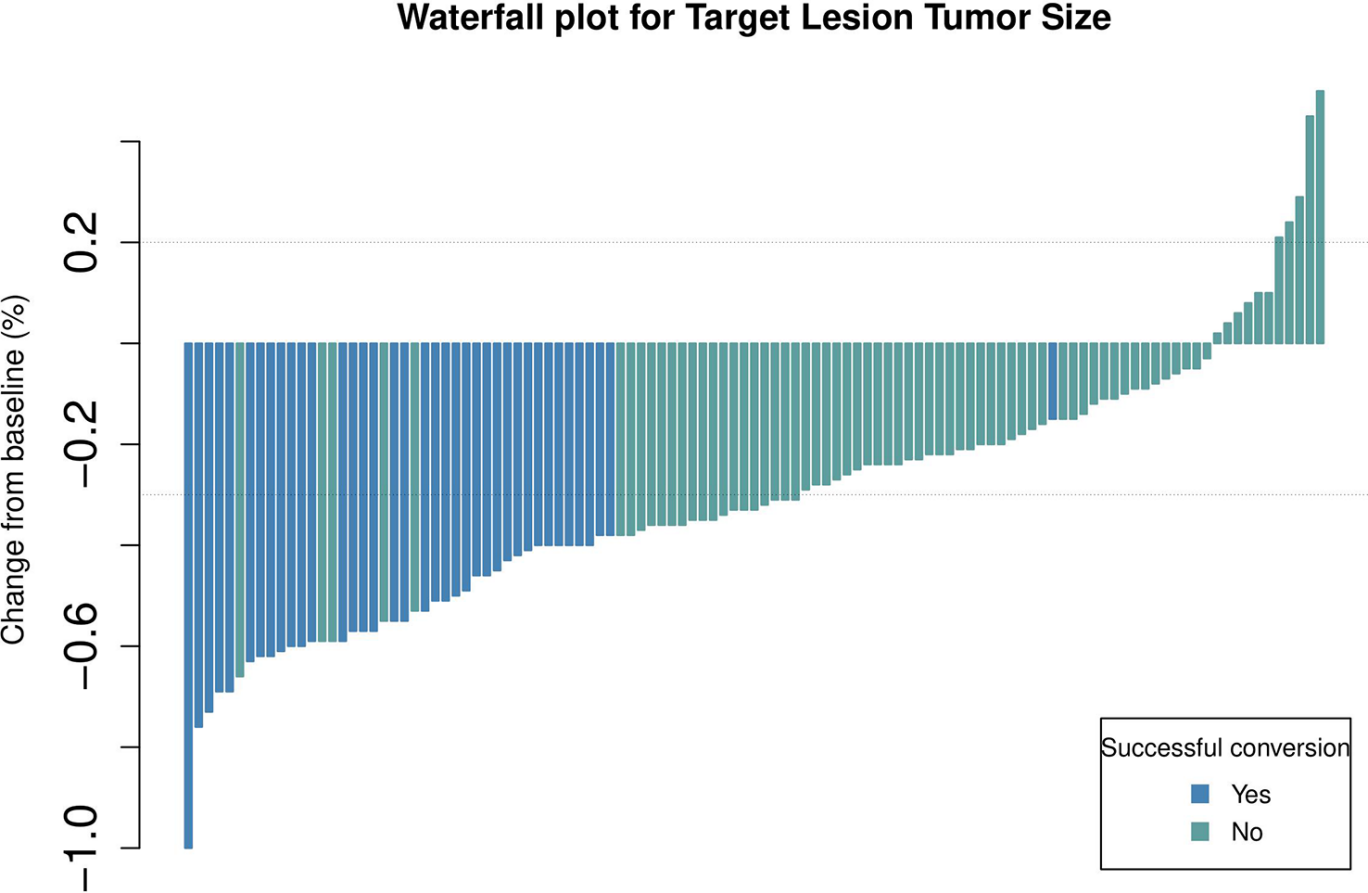
Waterfall plot demonstrating the target lesion size estimated using mRECIST guidelines.

Short-term curative effects evaluated based on the mRECIST guidelines (8, 20, 21). The maximum therapeutic response are significantly correlated with the prognosis (22). Because the patients included in this study had a high tumour burden, we evaluated the maximum tumour response to treatment based on the mRECIST guidelines. Waterfall plots demonstrating the maximum tumour response are depicted in **Figure 2**. The ORR and DCR of patients were 54.1% and 94.6%, respectively. The ORR was higher in the conversion surgery group (successful vs unsuccessful: 65% versus 36%, p = 0.08).

**Figure 3:**
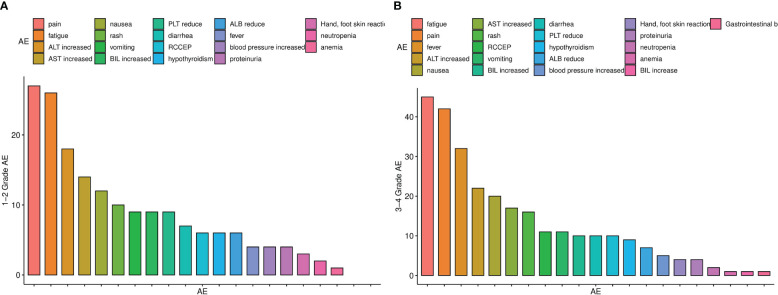
**(A)** Histogram of adverse reactions of grades 1–2, **(B)** Histogram of adverse reactions of grades 3–4.

A total of 280 treatment-related AEs occurred during the follow-up period. The most common grade-1 and -2 AEs were abdominal pain, fatigue and abnormal liver function (**Figure 3A**). A total of 97 patients experienced at least 1 grade-3 or -4 AE (**Figure 3B**). The most common grade-3 and -4 AEs were fatigue, pain and fever. Additionally, a patient had gastrointestinal haemorrhage, which was successfully treated *via* endoscopic haemostasis.

### Prognostic analysis

**Figure 4:**
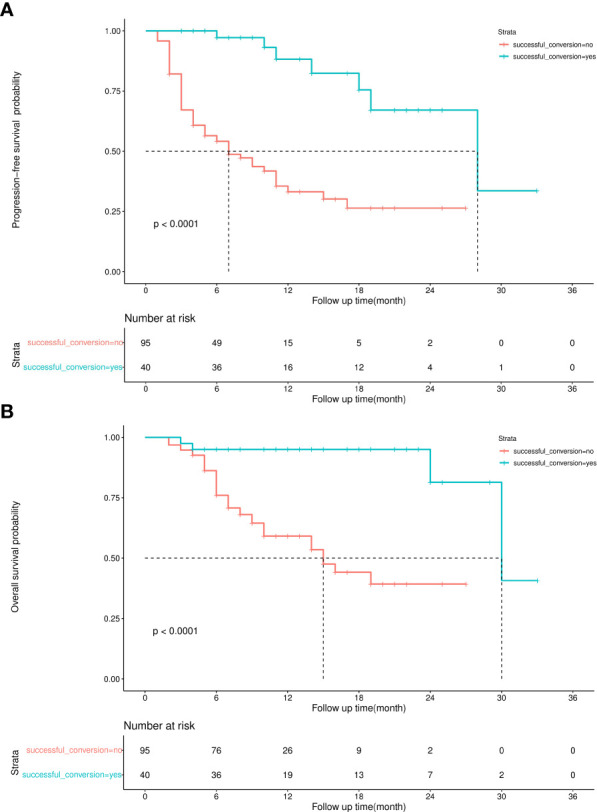
**(A)** Kaplan–Meier curve for progression-free survival, **(B)** Kaplan–Meier curve for overall survival.

**Figure 5:**
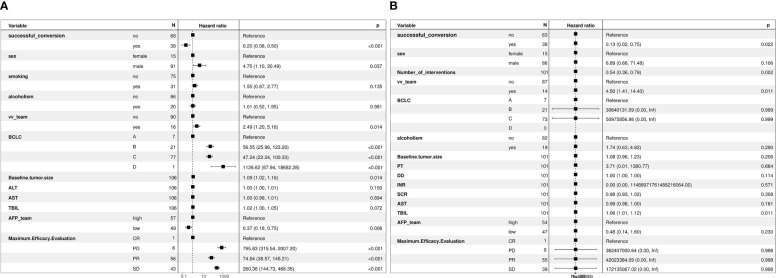
**(A)** Forest plot of multivariate Cox regression analysis of PFS, **(B)** Forest plot of multivariate Cox regression analysis of OS.

The median follow-up duration was 12 months and 11 months in the successful and unsuccessful conversion groups, respectively. The median PFS was 28 months and 7 months in the successful and unsuccessful conversion groups, respectively. The median OS was 30 months and 15 months in the successful and unsuccessful conversion groups, respectively. PFS and OS curves are shown in **Figures 4A, B**. As shown in the forest plot in **Figures 5A, B**, successful conversion surgery, sex, hapatic vein invasion, BCLC stage, baseline tumour size, AFP levels and maximum therapeutic response were independent prognostic factors for PFS. Multivariate Cox analysis showed that successful conversion surgery, number of interventions, hapatic vein invasion and total bilirubin levels were independent prognostic factors for OS.

### Sensitivity analysis

**Figure 6:**
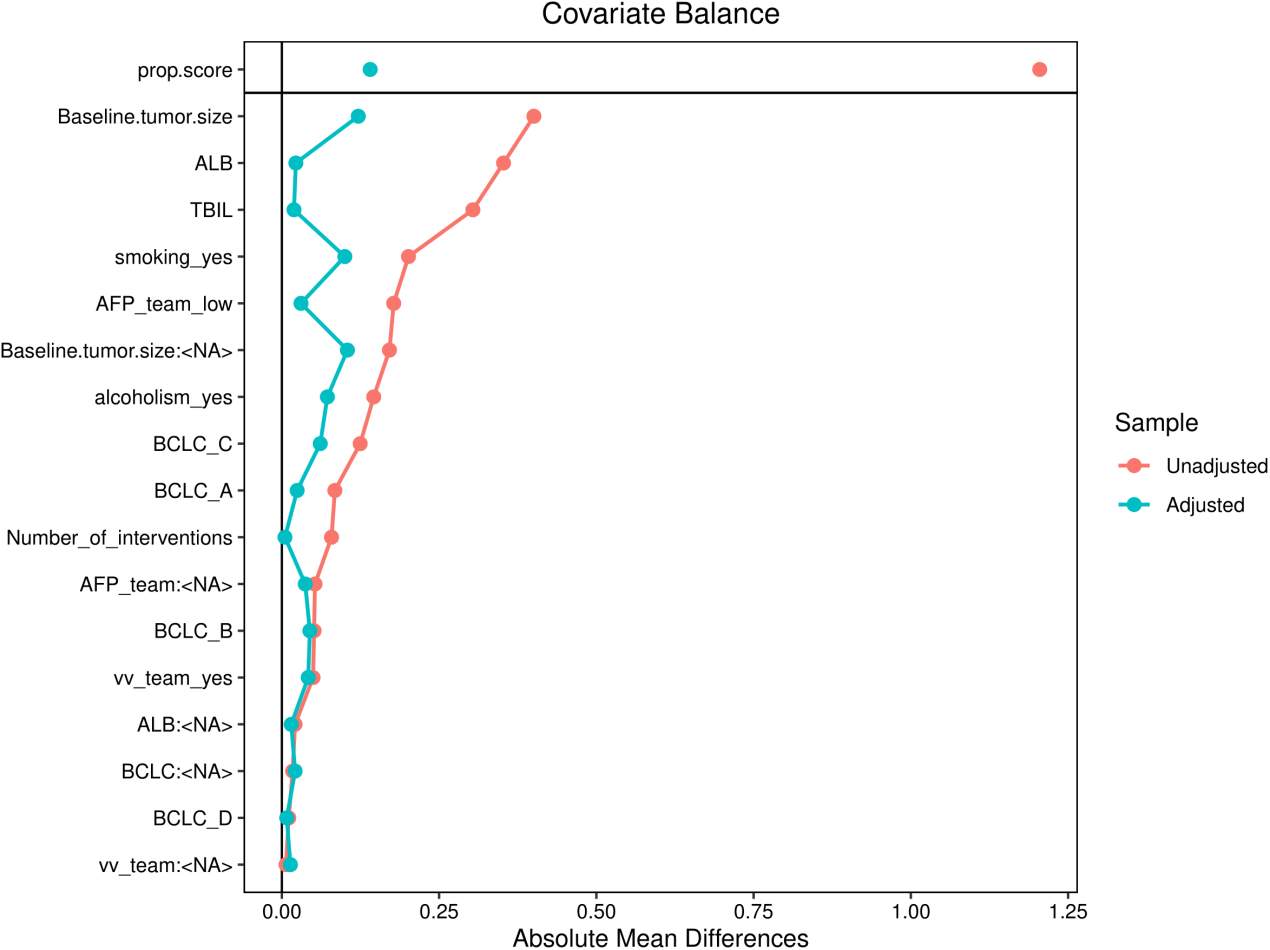
Love plot for standardised baseline differences before and after IPTW.

**Figure 7:**
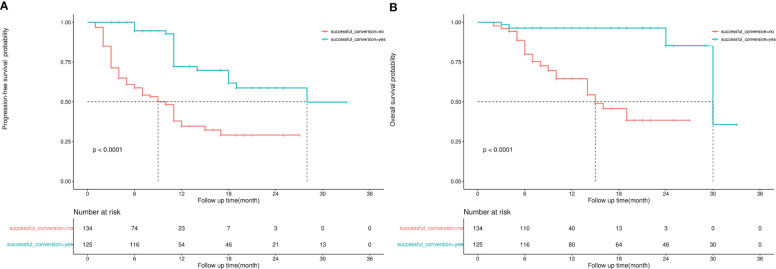
**(A)** IPW-adjusted Kaplan–Meier curve for PFS, **(B)** IPW-adjusted Kaplan–Meier curve for OS.

The sample size of this study is small, and the statistical power of multivariate analysis might have been inadequate. Therefore, IPW was used to balance confounding factors between the successful and unsuccessful conversion groups, and weighted survival analysis was subsequently used to examine sensitivity (23). As shown in **Figure 6**, the factors that may affect the prognosis of patients are incorporated into the IPTW equation. After IPTW analysis, no standardised differences exceeding the threshold were observed. IPW-adjusted survival curves were plotted to demonstrate the effects of successful conversion surgery on PFS and OS (**Figures 7A, B**). The final results showed that successful conversion surgery was an independent prognostic factor for both PFS and OS.

Because observational analyses have inherent limitations, the E-value was calculated to assess the sensitivity to unmeasured confounders (24). If the effect value of the unmeasured confounding factor reaches the E-value, the result is invalidated. The E-value of successful conversion surgery was 7.57 and 6.53 for OS and PFS, respectively, which indicates a relatively robust impact of successful conversion surgery on the prognosis of patients.

## Discussion

In this study, real-world data were used to evaluate the effectiveness of HAIC combined with immunotherapy and molecular targeted therapy in patients with primary uHCC. Patients with high tumour burden had a high tumour regression rate after combination therapy, and successful conversion surgery had strong protective effects on the prognosis. Although the incidence of side effects owing to combination therapy is high, they can be well managed. To adjust for possible confounding factors, the article employs inverse probability weighting. In addition, we calculated E-values, which allow us to assess the effect of unmeasured confounders on the final results in a quantitative manner. The experimental design and methodology of this study will assist us in evaluating the causal relationship between HAIC combined with systemic therapy and patient prognosis, despite the fact that it is a real-world study.

Because most patients with HCC are diagnosed at a middle or advanced stage, they are ineligible for radical local treatment. Before immunotherapy was introduced, TACE was the standard treatment option for middle-stage liver cancer, whereas molecular targeted therapy (sorafenib or lenvatinib) was the standard treatment for advanced HCC (3).

In recent years, several novel systemic therapies have been introduced for the treatment of advanced HCC. In the IMbrave 150 study, combination therapy with atezolizumab and bevacizumab was identified as a better treatment option than sorafenib monotherapy for uHCC (4). In the ORIENT-32 trial, the effectiveness of combination therapy with sintilimab and a bevacizumab biosimilar (IBI305) was found to be superior to that of sorafenib monotherapy, which has been approved by the FDA for the first-line treatment of advanced unresectable liver disease (25). The phase III HIMALAYA trial demonstrated that combined with sorafenib monotherapy, durvalumab plus tremelimumab pre-stimulation treatment significantly improved survival (26). At present, the combination of molecular targeted drugs and immune checkpoint inhibitors is the first-line treatment for advanced liver cancer.

With the advancement of systemic therapy, the use of TACE therapy for treating HCC has reduced. Systemic therapy is recommended for patients with diffuse stage-B liver cancer (BCLC 2022) (27). Before immunotherapy was introduced, significant attempts were made to combine TACE and TKI drugs to improve therapeutic effects and prognosis. However, the results were contradictory (28–30). We speculate that the reason for this phenomenon may be the poor efficacy of TACE therapy in patients with a high tumour burden. However, TKI drugs are only effective in patients with a high tumour burden (7, 8, 31).

HAIC is more effective than TACE in patients with a high tumour burden (10, 32, 33). The combination of HAIC and molecular targeted therapy can increase the tumour regression rate and improve prognosis (13, 14). The findings of this study are similar to those of previous related studies. As evaluated based on the mRECIST guidelines, the overall ORR and DCR were 54.1% and 94.6%, respectively. These rates might have been overestimated because 24 patients had imaging data but cannot be judged. However, the results of survival analysis indicated that combination therapy was associated with a positive outcome. The overall median PFS and OS were 12 months and 30 months, respectively, which are higher than those reported in the IMBRAVE 150 and LEAP-002 studies (4, 34). Therefore, combination therapy may be more effective for patients with a high tumour burden.

At least one side effect was experienced by almost all patients. A total of 97 (72%) patients experienced AEs of grades 3–4. Fatigue, pain and fever were the most common grade-3 and -4 AEs. In addition, a patient had gastrointestinal haemorrhage, which was successfully treated *via* endoscopic haemostasis. The incidence of grade-3 and -4 AEs was significantly higher in this study than in previous studies (14, 35). This increase may be attributed to the inclusion of adverse reactions related to the process of perfusion therapy, such as abdominal pain and fever, in this study. Although these adverse reactions are graded high according to CTCAE 4.0 (36), they are manageable and have a minimal impact on the outcome of patients. No unique adverse effects associated with combination therapy were observed. The common AEs observed in this study are manageable despite their high incidence.

Furthermore, the prognostic impact of successful conversion surgery was assessed. Of the 135 patients with primary uHCC, 40 patients were successfully treated with surgery after the completion of combination therapy. The rate of surgical conversion in patients with uHCC after systemic and local therapy varies widely (35, 37). Owing to the small sample size of previous studies, there is a greater possibility of selection bias. To the best of our knowledge, this study employed the largest cohort to evaluate the efficacy of HAIC combined with immunotherapy and targeted therapy in uHCC. The surgical conversion rate of patients in this study is considered significant for future investigation into this field. Multivariate Cox, IPTW and E-value revealed that successful conversion surgery was an independent prognostic factor in uHCC. This finding indicates that surgical treatment should be administered to eligible patients with primary unresectable liver cancer to improve their prognosis.

This retrospective study has several limitations. First, HAIC combined with immunotherapy and targeted therapy has not been used in clinical practice for a long time, and the overall follow-up period was short. Further follow-up is required to establish long-term efficacy and side effects. Second, this study had a retrospective design with an unavoidable bias, such as selection bias in the inclusion of patients and information bias in the evaluation of imaging data. Due to being a retrospective study and a single-center study, the collected data is somewhat limited. For example, Previous studies showed that the nutritional state of HCC patients has a great impact on prognosis (38). In our study, we did not gather information about these factors. Additionally, because the data were collected from regional cancer centres, the results may not apply to all primary care units. It is necessary to conduct prospective multicentre studies to verify the results of this study. According to our study, the short-term curative effect of patients is greater than that of previous studies and randomized controlled trials. Despite the favorable short-term efficacy of combination therapy, selection bias may have contributed to this high efficacy.

In conclusion, HAIC combined with immunotherapy and molecular targeted therapy has a higher tumour regression rate and surgical conversion rate in patients with primary unresectable liver cancer, and the side effects of this combination therapy are well manageable. Patients undergoing surgery after combination therapy have survival benefits.

## Data availability statement

The raw data supporting the conclusions of this article will be made available by the authors, without undue reservation.

## Ethics statement

Written informed consent was obtained from the individual(s), and minor(s)’ legal guardian/next of kin, for the publication of any potentially identifiable images or data included in this article.

## Author contributions

Conception and design: WX and HY; acquisition and analysis of data: WZ and CL; methodology and soft-ware: KZ and ML; formal analysis: XY; data curation and radiological evaluation: QZ and WG; manuscript writing: WZ and KZ; writing – review and editing: DL, HM and HL; supervision: ZG. All authors contributed to the article and approved the submitted version.

## References

[1] Bray F Ferlay J Soerjomataram I Siegel RL Torre LA Jemal A. Global cancer statistics 2018: GLOBOCAN estimates of incidence and mortality worldwide for 36 cancers in 185 countries. CA Cancer J Clin (2018) 68(6):394–424. doi: 10.3322/caac.21492 30207593

[2] McGlynn KA Petrick JL El-Serag HB. Epidemiology of hepatocellular carcinoma. Hepatology (2021) 73 Suppl 1(Suppl 1):4–13. doi: 10.1002/hep.31288 PMC7577946 32319693

[3] Llovet JM De Baere T Kulik L Haber PK Greten TF Meyer T. Locoregional therapies in the era of molecular and immune treatments for hepatocellular carcinoma. Nat Rev Gastroenterol Hepatol (2021) 18(5):293–313. doi: 10.1038/s41575-020-00395-0 33510460

[4] Finn RS Qin S Ikeda M Galle PR Ducreux M Kim TY. Atezolizumab plus bevacizumab in unresectable hepatocellular carcinoma. N Engl J Med (2020) 382(20):1894–905. doi: 10.1056/NEJMoa1915745 32402160

[5] Reig M Forner A Rimola J Ferrer-Fàbrega J Burrel M Garcia-Criado Á. BCLC strategy for prognosis prediction and treatment recommendation: The 2022 update. J Hepatol (2022) 76(3):681–93. doi: 10.1016/j.jhep.2021.11.018 PMC8866082 34801630

[6] Llovet JM Villanueva A Marrero JA Schwartz M Meyer T Galle PR. Trial design and endpoints in hepatocellular carcinoma: AASLD consensus conference. Hepatology (2021) 73 Suppl 1:158–91. doi: 10.1002/hep.31327 32430997

[7] Wang Q Xia D Bai W Wang E Sun J Huang M. Development of a prognostic score for recommended TACE candidates with hepatocellular carcinoma: A multicentre observational study. J Hepatol (2019) 70(5):893–903. doi: 10.1016/j.jhep.2019.01.013 30660709

[8] Han G Berhane S Toyoda H Bettinger D Elshaarawy O Chan A. Prediction of survival among patients receiving transarterial chemoembolization for hepatocellular carcinoma: A response-based approach. Hepatology (2020) 72(1):198–212. doi: 10.1002/hep.31022 31698504 PMC7496334

[9] Li S Xu J Zhang H Hong J Si Y Yang T. The role of hepatic arterial infusion chemotherapy in the treatment of hepatocellular carcinoma: A systematic review and meta-analysis. Chemotherapy (2021) 66(4):124–33. doi: 10.1159/000518257 34515082

[10] Li QJ He MK Chen HW Fang WQ Zhou YM Xu L. Hepatic arterial infusion of oxaliplatin, fluorouracil, and leucovorin versus transarterial chemoembolization for Large hepatocellular carcinoma: A randomized phase III trial. J Clin Oncol (2022) 40(2):150–60. doi: 10.1200/JCO.21.00608 34648352

[11] Li S Mei J Wang Q Shi F Liu H Zhao M. Transarterial infusion chemotherapy with FOLFOX for advanced hepatocellular carcinoma: a multi-center propensity score matched analysis of real-world practice. Hepatobiliary Surg Nutr (2021) 10(5):631–45. doi: 10.21037/hbsn.2020.03.14 PMC8527433 34760967

[12] He M Li Q Zou R Shen J Fang W Tan G. Sorafenib plus hepatic arterial infusion of oxaliplatin, fluorouracil, and leucovorin vs sorafenib alone for hepatocellular carcinoma with portal vein invasion: A randomized clinical trial. JAMA Oncol (2019) 5(7):953–60. doi: 10.1001/jamaoncol.2019.0250 PMC6512278 31070690

[13] Zheng K Zhu X Fu S Cao G Li WQ Xu L. Sorafenib plus hepatic arterial infusion chemotherapy versus sorafenib for hepatocellular carcinoma with major portal vein tumor thrombosis: A randomized trial. Radiology (2022) 303(2):455–64. doi: 10.1148/radiol.211545 35103539

[14] Xin Y Cao F Yang H Zhang X Chen Y Cao X. Efficacy and safety of atezolizumab plus bevacizumab combined with hepatic arterial infusion chemotherapy for advanced hepatocellular carcinoma. Front Immunol (2022) 13:929141. doi: 10.3389/fimmu.2022.929141 35990634 PMC9388744

[15] He MK Liang RB Zhao Y Xu YJ Chen HW Zhou YM. Lenvatinib, toripalimab, plus hepatic arterial infusion chemotherapy versus lenvatinib alone for advanced hepatocellular carcinoma. Ther Adv Med Oncol (2021) 13:17588359211002720. doi: 10.1177/17588359211002720 PMC8010824 33854567

[16] Malik AY Foster C. The revised Declaration of Helsinki: cosmetic or real change. J R Soc Med (2016) 109(5):184–9. doi: 10.1177/0141076816643332 PMC4872207 27150712

[17] Lencioni R Llovet JM. Modified RECIST (mRECIST) assessment for hepatocellular carcinoma. Semin Liver Dis (2010) 30(1):52–60. doi: 10.1055/s-0030-1247132 20175033 PMC12268942

[18] Austin PC. An Introduction to Propensity Score Methods for Reducing the Effects of Confounding in Observational Studies. Multivariate Behav Res (2011) 46(3):399–424. doi: 10.1080/00273171.2011.568786 PMC3144483 21818162

[19] VanderWeele TJ Ding P. Sensitivity analysis in observational research: Introducing the e-value. Ann Intern Med (2017) 167(4):268–74. doi: 10.7326/M16-2607 28693043

[20] Llovet JM Lencioni R. mRECIST for HCC: Performance and novel refinements. J Hepatol (2020) 72(2):288–306. doi: 10.1016/j.jhep.2019.09.026 31954493 PMC12452114

[21] Lencioni R Montal R Torres F Park JW Decaens T Raoul JL. Objective response by mRECIST as a predictor and potential surrogate end-point of overall survival in advanced HCC. J Hepatol (2017) 66(6):1166–72. doi: 10.1016/j.jhep.2017.01.012 28131794

[22] Xia D Wang Q Bai W Wang E Wang Z Mu W. Optimal time point of response assessment for predicting survival is associated with tumor burden in hepatocellular carcinoma receiving repeated transarterial chemoembolization. Eur Radiol (2022) 32(9):5799–810. doi: 10.1007/s00330-022-08716-4 35381853

[23] Huang LL Wei YY Chen F. [Confounder adjustment in observational comparative effectiveness researches: (1) statistical adjustment approaches for measured confounder]. Zhonghua Liu Xing Bing Xue Za Zhi (2019) 40(10):1304–9. doi: 10.3760/cma.j.issn.0254-6450.2019.10.024 31658535

[24] Huang LL Wei YY Chen F. [Confounder adjustment in observational comparative effectiveness researches: (2) statistical adjustment approaches for unmeasured confounders]. Zhonghua Liu Xing Bing Xue Za Zhi (2019) 40(11):1450–5. doi: 10.3760/cma.j.issn.0254-6450.2019.11.020 31838820

[25] Ren Z Xu J Bai Y Xu A Cang S Du C. Sintilimab plus a bevacizumab biosimilar (IBI305) versus sorafenib in unresectable hepatocellular carcinoma (ORIENT-32): a randomised, open-label, phase 2-3 study. Lancet Oncol (2021) 22(7):977–90. doi: 10.1016/S1470-2045(21)00252-7 34143971

[26] Kudo M. Durvalumab Plus Tremelimumab: A Novel Combination Immunotherapy for Unresectable Hepatocellular Carcinoma. Liver Cancer (2022) 11(2):87–93. doi: 10.1159/000523702 PMC9109076 35634425

[27] Reig M Forner A Rimola J Ferrer-Fábrega J Burrel M Garcia-Criado A. BCLC strategy for prognosis prediction and treatment recommendation: The 2022 update. J Hepatol (2022) 76(3):681–93. doi: 10.1016/j.jhep.2021.11.018 PMC8866082 34801630

[28] Kudo M Imanaka K Chida N Nakachi K Tak WY Takayama T. Phase III study of sorafenib after transarterial chemoembolisation in Japanese and Korean patients with unresectable hepatocellular carcinoma. Eur J Cancer (2011) 47(14):2117–27. doi: 10.1016/j.ejca.2011.05.007 21664811

[29] Park JW Kim YJ Kim DY Bae SH Paik SW Lee YJ. Sorafenib with or without concurrent transarterial chemoembolization in patients with advanced hepatocellular carcinoma: The phase III STAH trial. J Hepatol (2019) 70(4):684–91. doi: 10.1016/j.jhep.2018.11.029 30529387

[30] Peng Z Fan W Zhu B Wang G Sun J Xiao C. Lenvatinib combined with transarterial chemoembolization as first-line treatment for advanced hepatocellular carcinoma: A phase III, randomized clinical trial (LAUNCH). J Clin Oncol (2023) 41(1):117–27. doi: 10.1200/JCO.22.00392 35921605

[31] Chen M Cao J Hu J Topatana W Li S Juengpanich S. Clinical-radiomic analysis for pretreatment prediction of objective response to first transarterial chemoembolization in hepatocellular carcinoma. Liver Cancer (2021) 10(1):38–51. doi: 10.1159/000512028 33708638 PMC7923935

[32] Lyu N Lin Y Kong Y Zhang Z Liu L Zheng L. FOXAI: a phase II trial evaluating the efficacy and safety of hepatic arterial infusion of oxaliplatin plus fluorouracil/leucovorin for advanced hepatocellular carcinoma. Gut (2018) 67(2):395–6. doi: 10.1136/gutjnl-2017-314138 28592441

[33] Ueshima K Ogasawara S Ikeda M Yasui Y Terashima T Yamashita T. Hepatic arterial infusion chemotherapy versus sorafenib in patients with advanced hepatocellular carcinoma. Liver Cancer (2020) 9(5):583–95. doi: 10.1159/000508724 PMC7548914 33083282

[34] Finn RS Kudo M Merle P Meyer T Qin S Ikeda M. LBA34 primary results from the phase III LEAP-002 study: Lenvatinib plus pembrolizumab versus lenvatinib as first-line (1L) therapy for advanced hepatocellular carcinoma (aHCC). Ann OF Oncol (2022) 33:S1401. doi: 10.1016/j.annonc.2022.08.031

[35] Zhang J Zhang X Mu H Yu G Xing W Wang L. Surgical conversion for initially unresectable locally advanced hepatocellular carcinoma using a triple combination of angiogenesis inhibitors, anti-PD-1 antibodies, and hepatic arterial infusion chemotherapy: A retrospective study. Front Oncol (2021) 11:729764. doi: 10.3389/fonc.2021.729764 34868921 PMC8632765

[36] Chen AP Setser A Anadkat MJ Cotliar J Olsen EA Garden BC. Grading dermatologic adverse events of cancer treatments: the common terminology criteria for adverse events version 4.0. J OF THE Am Acad OF Dermatol (2012) 67(5):1025–39. doi: 10.1016/j.jaad.2012.02.010 22502948

[37] Zhu XD Huang C Shen YH Ji Y Ge NL Qu XD. Downstaging and resection of initially unresectable hepatocellular carcinoma with tyrosine kinase inhibitor and anti-PD-1 antibody combinations. Liver Cancer (2021) 10(4):320–9. doi: 10.1159/000514313 PMC8339461 34414120

[38] Rong W Xia H Zhang K Zhang Y Tao C Wu F. Serum metabolic effects of corn oligopeptides with 7-day supplementation on early post-surgery primary liver cancer patients: a double-blind randomized controlled trial. Hepatobiliary Surg Nutr (2022) 11(6):834–47. doi: 10.21037/hbsn-21-116 PMC9745621 36523946

