# Supplementary material for: Hepatic arterial infusion chemotherapy combined with anti-PD-1/PD-L1 immunotherapy and molecularly targeted agents for advanced hepatocellular carcinoma: a real world study
Source: Front Immunol. 2023 Apr 26;14:1127349. doi: 10.3389/fimmu.2023.1127349 (PMC10169627; doi:10.3389/fimmu.2023.1127349)
Supplement: Supplementary file 1 [file DataSheet_1.docx]

Summary descriptives table by groups of ‘successful_conversion’

|  | **[ALL]** | **no** | **yes** | **p.overall** |
| --- | --- | --- | --- | --- |
|  | ***N=135*** | ***N=95*** | ***N=40*** |  |
| sex: |  |  |  | 0.848 |
| female | 24 (17.8%) | 16 (16.8%) | 8 (20.0%) |  |
| male | 111 (82.2%) | 79 (83.2%) | 32 (80.0%) |  |
| age | 58.0 [51.0;64.5] | 57.0 [50.0;64.0] | 59.0 [54.0;66.2] | 0.240 |
| Number_of_interventions | 3.00 [2.00;3.00] | 2.00 [1.00;3.50] | 3.00 [2.00;3.00] | 0.311 |
| targeted_drug: |  |  |  | 0.112 |
| Apatinib | 5 (3.70%) | 2 (2.11%) | 3 (7.50%) |  |
| Bevacizumab | 57 (42.2%) | 36 (37.9%) | 21 (52.5%) |  |
| Lenvatinib | 67 (49.6%) | 52 (54.7%) | 15 (37.5%) |  |
| Sorafenib | 6 (4.44%) | 5 (5.26%) | 1 (2.50%) |  |
| ICI: |  |  |  | 0.460 |
| Atezolizumab | 1 (0.74%) | 1 (1.05%) | 0 (0.00%) |  |
| Camrelizumab | 31 (23.0%) | 24 (25.3%) | 7 (17.5%) |  |
| Pembrolizumab | 1 (0.74%) | 1 (1.05%) | 0 (0.00%) |  |
| Sintilimab | 96 (71.1%) | 65 (68.4%) | 31 (77.5%) |  |
| Tislelizumab | 3 (2.22%) | 3 (3.16%) | 0 (0.00%) |  |
| Toripalimab | 3 (2.22%) | 1 (1.05%) | 2 (5.00%) |  |
| hypertension: |  |  |  | 0.853 |
| no | 101 (74.8%) | 72 (75.8%) | 29 (72.5%) |  |
| yes | 34 (25.2%) | 23 (24.2%) | 11 (27.5%) |  |
| diabetes: |  |  |  | 0.777 |
| no | 119 (88.1%) | 83 (87.4%) | 36 (90.0%) |  |
| yes | 16 (11.9%) | 12 (12.6%) | 4 (10.0%) |  |
| heart.disease: |  |  |  | 0.669 |
| no | 129 (95.6%) | 90 (94.7%) | 39 (97.5%) |  |
| yes | 6 (4.44%) | 5 (5.26%) | 1 (2.50%) |  |
| smoking: |  |  |  | 0.028 |
| no | 99 (73.3%) | 64 (67.4%) | 35 (87.5%) |  |
| yes | 36 (26.7%) | 31 (32.6%) | 5 (12.5%) |  |
| alcoholism: |  |  |  | 0.075 |
| no | 111 (82.2%) | 74 (77.9%) | 37 (92.5%) |  |
| yes | 24 (17.8%) | 21 (22.1%) | 3 (7.50%) |  |
| Liver.cancer.etiology: |  |  |  | 0.278 |
| alchol | 1 (0.74%) | 0 (0.00%) | 1 (2.50%) |  |
| HBV | 126 (93.3%) | 90 (94.7%) | 36 (90.0%) |  |
| HCV | 8 (5.93%) | 5 (5.26%) | 3 (7.50%) |  |
| vv_team: |  |  |  | 0.634 |
| no | 113 (86.3%) | 78 (84.8%) | 35 (89.7%) |  |
| yes | 18 (13.7%) | 14 (15.2%) | 4 (10.3%) |  |
| vp: |  |  |  | 0.489 |
| 0 | 54 (41.2%) | 37 (40.2%) | 17 (43.6%) |  |
| 2 | 6 (4.58%) | 3 (3.26%) | 3 (7.69%) |  |
| 3 | 29 (22.1%) | 23 (25.0%) | 6 (15.4%) |  |
| 4 | 42 (32.1%) | 29 (31.5%) | 13 (33.3%) |  |
| BCLC: |  |  |  | 0.204 |
| A | 9 (6.92%) | 4 (4.40%) | 5 (12.8%) |  |
| B | 22 (16.9%) | 14 (15.4%) | 8 (20.5%) |  |
| C | 98 (75.4%) | 72 (79.1%) | 26 (66.7%) |  |
| D | 1 (0.77%) | 1 (1.10%) | 0 (0.00%) |  |
| Baseline.tumor.size | 8.95 [6.27;13.0] | 10.0 [7.03;14.0] | 7.95 [5.38;10.7] | 0.045 |
| Maximum.Efficacy.Evaluation: |  |  |  | 0.089 |
| CR | 1 (0.90%) | 0 (0.00%) | 1 (1.37%) |  |
| PD | 6 (5.41%) | 6 (8.22%) | 0 (0.00%) |  |
| PR | 59 (53.2%) | 34 (46.6%) | 25 (65.8%) |  |
| SD | 45 (40.5%) | 32 (43.8%) | 13 (34.2%) |  |
| Hb | 139 [124;152] | 137 [123;151] | 142 [129;156] | 0.242 |
| WBC | 5.36 [4.17;6.88] | 5.29 [3.96;7.18] | 5.59 [4.51;6.39] | 0.810 |
| NE | 3.49 [2.43;4.49] | 3.49 [2.45;4.66] | 3.34 [2.42;4.48] | 0.844 |
| PLT | 165 [118;243] | 163 [118;230] | 170 [117;247] | 0.816 |
| PT | 12.3 [11.7;13.1] | 12.5 [11.7;13.2] | 12.1 [11.7;12.8] | 0.203 |
| APTT | 27.7 [25.6;30.0] | 27.9 [25.9;30.3] | 27.5 [25.1;29.5] | 0.326 |
| DD | 949 [500;1903] | 982 [505;2119] | 779 [345;1476] | 0.101 |
| INR | 1.07 [1.02;1.15] | 1.10 [1.02;1.16] | 1.06 [1.02;1.12] | 0.272 |
| GLU | 5.12 [4.53;5.93] | 5.18 [4.53;6.07] | 5.07 [4.58;5.49] | 0.874 |
| SCR | 64.0 [56.5;76.0] | 64.0 [56.5;75.5] | 67.0 [56.5;76.0] | 0.320 |
| ALB | 39.1 [35.6;42.0] | 38.4 [35.1;41.8] | 40.5 [37.5;42.6] | 0.024 |
| ALT | 32.0 [20.0;52.0] | 33.0 [20.0;52.0] | 32.0 [19.2;49.2] | 0.544 |
| AST | 52.0 [34.0;85.0] | 59.0 [35.0;92.0] | 45.0 [33.8;65.2] | 0.104 |
| TBIL | 17.1 [12.1;23.7] | 18.2 [12.2;25.2] | 15.1 [11.4;21.3] | 0.117 |
| AFP_team: |  |  |  | 0.092 |
| high | 68 (52.3%) | 52 (57.8%) | 16 (40.0%) |  |
| low | 62 (47.7%) | 38 (42.2%) | 24 (60.0%) |  |
